# PLT Counts as a Predictive Marker after Plasma Exchange in Patients with Hepatitis B Virus-Related Acute-on-Chronic Liver Failure

**DOI:** 10.3390/jcm12030851

**Published:** 2023-01-20

**Authors:** Xue Li, Hao Li, Yucui Zhu, Huaqian Xu, Shanhong Tang

**Affiliations:** 1 Department of Gastroenterology, The General Hospital of Western Theater Command, Chengdu 610083, China; 2 Clinic, The General Hospital of Western Theater Command, Chengdu 610083, China

**Keywords:** HBV-ACLF, PLT, MELD, PE

## Abstract

Background and Objectives: The platelet (PLT) value in hepatitis B-related acute-on-chronic liver failure (HBV-ACLF) is not sufficiently understood. The present study aimed to evaluate the prognostic effect of PLT on the prediction of HBV-ACLF outcomes after plasma exchange (PE). Methods: HBV-ACLF patients treated with PE between January 2017 and August 2021 were followed up for at least 6 months. Cox regression was performed to develop the predictive model, and the model’s performance was analyzed using the receiver operating characteristic curve (ROC). Results: A total of 170 patients were included. The overall survival rate within 180 days was 75.88%. Age, PLT, total bilirubin (TBil), and the iMELD scores were independent risk factors affecting the prognosis of HBV-ACLF patients after PE. According to the Cox regression results, the new model was calculated: R = 0.142 × iMELD-0.009 × PLT. The area under the curve (AUC) of the receiver operating characteristic curve (ROC) was 0.758 (95% CI 0.678–0.838), and patients with lower PLT-iMELD scores (<4.50) had a better prognosis (*p* < 0.001). Conclusion: PLT is a valuable prognostic biomarker for HBV-ACLF patients after PE. The modified iMELD model incorporating PLT has a better sensitivity and efficacy in predicting the prognosis of patients.

## 1. Introduction

Acute-on-chronic liver failure (ACLF) is an acute liver injury syndrome based on chronic liver disease, with rapid progression and high short-term mortality [1]. The etiology of ACLF is mainly alcoholic cirrhosis in Western countries, and hepatitis B virus (HBV) infection is the main cause in the Asia-Pacific region [2,3]. According to previous studies [4], the 28-day transplant-free survival rate of ACLF patients was 68%, and the 90-day transplant-free survival rate was only 50.2%. At present, the treatment of ACLF includes standard medical treatment (SMT), the artificial liver support system (ALSS), and liver transplantation (LT), among which LT is the most effective treatment. However, the shortage of donor livers, high surgical cost, and postoperative immune rejection limit the clinical application of liver transplantation. ALSS is an in vitro support technique that plays an important role in the treatment of ACLF. Among the many models of ALSS, plasma exchange (PE) is widely used in the Asia-Pacific region. PE can remove toxins, reduce inflammation, promote liver repair, and improve the survival of HBV-ACLF patients [5]. Liver regeneration as an important step in the reversal of liver injury, rendering ALSS an effective bridging therapy for LT or hepatocyte self-regeneration in patients with ACLF. The combination of ALSS and LT therapy improves short-term survival among patients with HBV-ACLF, and ALSS treatment before LT was found to be an independent protective factor for 4-week survival after LT [6].

A variety of models have been used to evaluate the prognosis of ACLF, but most of them mainly consider liver reserve function and multi-organ failure, while few focus on liver tissue repair and regeneration ability, which is the key to improving damaged liver function. At present, the clinical value of alpha-fetoprotein (AFP) in the prognosis of liver failure has attracted great attention [7]. In addition to the function of coagulation, PLT also has a certain value in liver regeneration. Recent studies [8] have shown that platelets are rich in many growth factors related to the induction of liver regeneration, indicating that PLT participates in liver regeneration by interacting with hepatocytes and regulating the release of growth factors [9]. PLT mainly originates from mature megakaryocytes in the bone marrow, 2/3 of which are distributed in the peripheral circulation blood and 1/3 of which are stored in the liver and spleen. PLT production is mainly regulated by thrombopoietin (TPO), while TPO is mainly synthesized in the liver. When liver failure occurs, TPO synthesis is affected, which further leads to a reduction in the platelet count [10]. The PLT to WBC ratio (PWR) has been reported to reflect a systemic inflammatory response. Studies [11] have shown that patients with ACLF have higher PWR levels, but the short-term prognosis of HBV-ACLF has not been formally determined. So far, the role of PLT in liver regeneration and its therapeutic effect has not been studied in depth. Therefore, it is important to explore more comprehensive, accurate, and convenient prognostic biomarkers for ACLF. In the comprehensive treatment of HBV-ACLF, we should pay close attention to patients’ PLT, AFP, and other regeneration indicators as far as possible to create an environment conducive to hepatocyte regeneration and strengthen the judgment of prognosis so as to improve the survival rate of patients. By collecting and analyzing the clinical data of HBV-ACLF patients treated with PE, this study aimed to construct a new, short-term prognosis model that considers the combination of liver regeneration and reserve capacity so as to provide rapid and accurate aid in clinical diagnosis and treatment.

## 2. Materials and Methods

### 2.1. Patients

In this study, we retrospectively collected ACLF patients treated with PE. A total of 226 ACLF patients treated with PE at the General Hospital of Western Theater Command from January 2017 to August 2021 were included in this study. The inclusion criteria were as follows: (1) ACLF was defined according to the 2019 APASL Consensus Recommendation [1]: acute liver injury in previously diagnosed or undiagnosed patients with chronic liver disease or cirrhosis, characterized by jaundice (serum bilirubin ≥ 5 mg/dL (≥85 μmol/L)) and coagulation dysfunction (INR ≥ 1.5 or prothrombin activity < 40%) and ascites and/or hepatic encephalopathy within 4 weeks of the disease course; (2) age over 18 years old, with no gender limit; and (3) HBV-ACLF patients treated with PE. The exclusion criteria were as follows: (1) other causes of ACLF, such as alcohol, HAV, HCV, and other forms of viral hepatitis; (2) the patient does not meet the APASL criteria; (3) combined with hepatocellular carcinoma (HCC); (4) the patient was recently taking antiplatelet drugs; (5) previous splenectomy was performed; (6) clinical data missing or patients lost to follow-up; and (7) LT therapy. The final cohort comprised 170 patients, including 150 males and 20 females, who were divided into a survival group and a non-survival group according to the 180-day survival status. The patients were followed up for 180 days by telephone and face-to-face interviews.

### 2.2. Observation Indicators

The age, gender, blood routine, liver and kidney function, coagulation parameters, and the MELD series scores of patients diagnosed with HBV-ACLF after PE were collected. Blood routine examination was performed using an automatic blood cell analyzer (model XN9100, Sysmex, Kobe, Japan). Liver function was detected by biochemical immunoassay (model DX1800, Beckman Coulter, Brea, California, USA), and the blood coagulation parameter was determined using an automatic hemagglutination instrument (model CS5100, Sysmex, Kobe, Japan). The PE volume was 1500–2000 mL/time, the replacement blood flow velocity was 80–120 mL/min, and the replacement time was 2–3 h. The blood pressure, heart rate, respiration, and body temperature were monitored throughout the replacement process, and the arterial and venous pressures were closely monitored.

### 2.3. Statistical Analysis

The results were expressed as the mean ± standard and median (P25, P75). Differences between the two unpaired groups were evaluated using the Mann–Whitney U test and Wilcoxon’s two-sample test. Receiver operating characteristic (ROC) curve analysis was performed. Pearson’s correlation was used to analyze the continuous variables. Univariate Cox regression was used to screen for risk factors, and independent risk factors were determined through multivariate Cox regression. A new prognostic scoring system was established based on the Cox proportional risk regression. Survival analysis was performed using Kaplan–Meier, and the differentiation analysis was evaluated by the log-rank test. The data were analyzed using SPSS 26.0 software (IBM Corporation, Somers, NY, USA). *p* < 0.05 was considered statistically significant.

## 3. Results

### 3.1. Characteristics and Outcomes of HBV-ACLF Patients after PE

A total of 170 HBV-ACLF patients who were treated with PE were enrolled in this study. In total, 56 patients were excluded for the following reasons: 10 cases of hepatocellular carcinoma, three cases of other tumors, 36 cases of other causes of ACLF, and seven cases of lost to follow-up. A total of 129 patients (129/170) survived and 41 patients (41/170) died within 180 days, and the 180-day survival rate was 75.88%. The PLT (113.00 (85.00, 154.00) vs. 93.00 (55.00, 93.00), *p* < 0.05) and AFP (88.58 (26.73, 260.05) vs. 48.07 (17.09, 141.82), *p* < 0.05) of the survival group were significantly higher than those of the non-survival group. The age, PT, INR, ALT, AST, TBil, MELD, MELD-Na, and iMELD of the survival group were significantly lower than those of the non-survival group (*p* < 0.05. There were no significant differences between groups in terms of gender, Alb, and Cre. Their baseline characteristics are shown in Table 1.

### 3.2. Correlation Analysis of PLT with Liver Function Parameters and the MELD Series Score

Pearson’s correlation analysis showed that PLT had no significant correlation with AST, TBil, AFP, PT, INR, MELD, or MELD-Na but was positively correlated with ALT and Alb (*p* < 0.05) and negatively correlated with iMELD (*p* < 0.05). The correlation between each parameter was represented by the *r*/*p* value. The correlation analysis is shown in Table 2.

### 3.3. Identification of Prognostic Risk Factors and Establishment of a New Prediction Model

**Figure 1:**
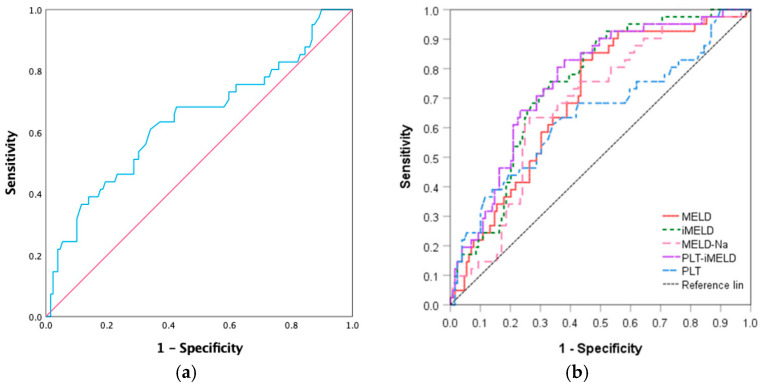
Efficacy of receiver operating characteristic curves (ROC) in predicting the outcome. (**a**) ROC curve for PLT counts. (**b**) ROC curve for the prognostic model. PLT: platelet; MELD: model for end-stage liver disease; MELD-Na: MELD-sodium; iMELD: integrated MELD; PLT-iMELD: platelet-integrated MELD.

The clinical variables were included in a univariate Cox analysis, and *p* < 0.05 was used to screen the risk factors. The results of the analysis showed that age, PLT, TBil, MELD, MELD-Na, and iMELD showed significant associations with the 180-day survival (*p* < 0.05). Then, the above six indicators were incorporated into a multivariate model, as shown in Table 3. The age (HR 1.044, 95%CI 1.012–1.077, *p* = 0.007), PLT (HR 0.990, 95%CI 0.984–0.997, *p* = 0.021), TBil (HR 1.002, 95%CI 1.000–1.004, *p* = 0.042) and iMELD (HR 1.037, 95%CI 1.016–1.059, *p* = 0.032) were independently associated with the prognosis of HBV-ACLF after PE over 180 days (Table 3). According to the method reported by Alsebaey et al. [12] and Qin et al. [13], a new model was established according to the β determination formula in the regression equation. Thus, a new prognostic model was generated: PLT-iMELD = 0.142 × iMELD-0.009 × PLT. The ROC curves for the PLT, MELD series scoring, and new model are shown in Figure 1. The improved iMELD prediction model shows better prediction values. The ROC analysis showed that the model has a good accuracy in predicting mortality based on PLT-iMELD (AUC = 0.758, 95%CI 0.678–0.838, *p* < 0.001), followed by iMELD (AUC = 0.745, 95%CI 0.666–0.823, *p* < 0.001), the MELD score (AUC = 0.695, 95%CI 0.609–0.782, *p* < 0.001), MELD-Na (AUC = 0.679, 95%CI 0.590–0.767, *p* < 0.001) score, and PLT (AUC = 0.643, 95%CI 0.539–0.747, *p* = 0.006). More details are displayed in Table 4.

### 3.4. Performance of the New Model

**Figure 2:**
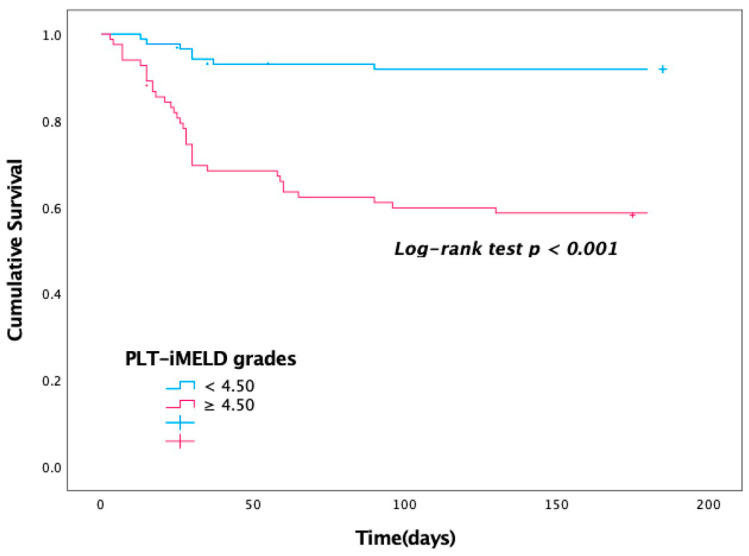
Kaplan–Meier curve for survival at 180 days among pediatric HBV-ACLF patients after PE. Kaplan–Meier curve for survival stratified by PLT-iMELD score < 4.50 and ≥4.50. PLT-iMELD: platelet-integrated MELD.

The newly established PLT-iMELD scoring model is effective in predicting the 180-day survival of HBV-ACLF patients after PE treatment. The cut-off value of 4.50, corresponding to the maximum value of Yoden’s index in the ROC analysis, was the critical value, the sensitivity of the model was 82.9%, and the specificity was 62.00%. The results showed that patients with higher PLT-iMELD scores (≥4.50) had an increased risk of poor outcomes. Thus, we further analyzed the survival of the patients according to their PLT-iMELD scores (Figure 2).

## 4. Discussion

HBV infection is the main cause of ACLF in the Asia-Pacific region. The affected patients have a rapid disease progression and high short-term mortality. The existing assessment models, including the MELD [14], MELD-Na [15], iMELD [16], CLIF, and AARC scores, play important roles in disease management and prognosis, but these models are based on organ failure and do not account for the indicators related to liver regeneration. Studies [17] have reported that PLT promotes liver regeneration, including direct action on the hepatocytes, synergistic action with hepatic sinusoidal endothelial cells, and synergistic action with Kupffer cells. Takahashi et al. [18] reviewed the role of PLT in promoting liver regeneration in hepatectomy and LT and discussed the prospect of using erythropoietin receptor agonists to promote liver regeneration. Padickakudy et al. [19] also found that 5-hydroxytryptamine (5-HT) in PLT may be a related promoter of human liver regeneration, suggesting that increasing the circulating platelets and 5-HT in the platelets may help to promote liver regeneration. This indicates a significant association between the 5-HT levels and early disease recurrence after hepatectomy. In addition, PLT has been related to the pathophysiology of infectious diseases, systemic inflammatory reactions, and immune diseases, and low PLT is related to disease severity [20].

At present, except for LT, all the existing treatments depend on the strong capacity for liver regeneration, which is the key to the prognosis of liver failure. Ling et al. [21] found that the use of ALSS as a bridging therapy for ACLF patients can reduce the MELD score to <30, which could improve the prognosis of ACLF patients, who were found to be similar to early liver transplantation patients. PE is conducive to liver function recovery and liver tissue regeneration, helping to create the conditions required for these processes. PE is widely used in China and other Asia-Pacific countries. It can remove toxic substances from the bodies of patients within the appropriate timeframe and reduce liver inflammation [22]. At the same time, it also supplements plasma proteins and coagulation factors, which can not only reduce edema and bleeding in patients but also reduce the chance of infection and facilitate the repair and regeneration of liver cells. Larsen et al. [23] showed that PE could stabilize the hemodynamic state of patients, improve the blood biochemical indexes, and improve the survival rate of ACLF patients. However, some studies [24] found that PE could not effectively remove a large number of water-soluble toxins distributed in the plasma; thus, it could not significantly improve the symptoms of severe hepatic encephalopathy. Although studies on liver regeneration after hepatectomy have been reported [25], studies on liver regeneration after PE treatment are still lacking. PE is an effective treatment for ACLF. When severe liver failure occurs, PE can be used to temporarily assist or replace the main function of the liver so as to provide a conducive environment for liver regeneration. Regarding liver regeneration in liver failure, previous studies [26] have shown that the expression of AFP can appear in the early stage of liver regeneration after partial liver resection. Wang et al. [27] speculated and, indeed, found that AFP could act as an independent predictor of ACLF prognosis. As a useful marker for predicting prognosis in HBV-ACLF, high AFP levels tend to suggest a better prognosis. In addition, our center [28] conducted a 3-month follow-up study on HBV-ACLF patients who received PE treatment and established an improved MELD model that includes the serum AFP levels. It was found that the new model combining these two independent factors had a better effect in predicting the prognosis of PE treatment. Due to the lack of a unified consensus on the global definition of ACLF and the etiological differences between the East and West, the existing models do not have a stable or perfect predictive power.

In this study, we found that PLT may be a prognostic marker for liver regeneration in HBV-ACLF patients treated with PE, and by combining PLT with the traditional models, we can effectively evaluate the liver conditions and prognosis from the perspective of liver regeneration. The new model is effective for the assessment of 180 days of survival and better than the MELD, MELD, and iMELD models. Patients with high PLT-iMELD scores (≥4.50) tend to have poor outcomes. The new model comprehensively evaluates regeneration and the injury. When the regeneration capacity is stronger than the injury, the prognosis of patients is better. However, when the injury is too extensive or the regeneration capacity of the liver is weakened, the prognosis of patients is worse. Therefore, a simple and effective prediction model is crucial for the judgment and prognosis of patients. The early detection parameters of prognosis and close observation of their changes, to a certain extent, can help clinical doctors to judge the degree of liver damage in patients, with a comprehensive evaluation of the patient’s restoration capacity and the reversibility of the injury, so as to adjust the treatment plan in a timely manner and improve the prognosis of HBV-ACLF patients. Some studies have suggested that treatment based on the direct binding of PLT counts could be used to induce liver regeneration in the future. However, the question of whether or not PLT can be used in the treatment of liver failure still requires further study.

There were also some limitations affecting our study. Firstly, it was a retrospective study, and the sample size was not large enough to represent the characteristics of patients with ACLF of different etiologies. Secondly, due to the lack of lactate and other serological indicators, other models were not included in the comparison. Thirdly, we did not carry out the dynamic detection of each indicator. In future studies, the serological indicators of a greater number of cases will be collected to dynamically evaluate the efficiency of each indicator model. Therefore, large-scale, multicenter, prospective studies are needed to evaluate the usability of this novel prognostic model.

## 5. Conclusions

PLT is a biomarker of liver regeneration after PE in HBV-ACLF patients. A Cox regression model was established to predict the 180-day prognosis of HBV-ACLF patients, which can be used as a tool to judge the prognosis of HBV-ACLF patients after PE treatment. The modified iMELD model incorporating PLT has a better sensitivity and efficacy in predicting the prognosis of patients. Therefore, in the course of disease diagnosis and treatment, it is equally important to pay attention to the liver regeneration ability and organ failure assessment.

## Figures and Tables

**Table 1:** Clinical characteristics and outcomes of HBV-ACLF patients.

Parameter	Survival (*n* = 129)	Non-Survival (*n* = 41)	*p* Value
Age (years)	42.80 ± 10.95	49.46 ± 8.38	0.000
Sex (M:F)	114/15	36/5	0.922
PLT (10^9^/L)	113.00 (85.00, 154.00)	93.00 (55.00, 93.00)	0.006
PT (s)	18.30 (15.60, 22.00)	21.10 (17.00, 25.00)	0.017
INR	1.60 (1.37, 1.97)	1.89 (1.52, 2.67)	0.011
AFP (ng/mL)	88.58 (26.73, 260.05)	48.07 (17.09, 141.82)	0.042
ALT (IU/L)	771.10 (219.05, 1658.65)	461.50 (150.50, 1440.25)	0.261
AST (IU/L)	436.20 (142.60, 1207.85)	376.20 (163.65, 1068.60)	0.736
Alb (g/L)	33.70 (31.50, 37.40)	32.60 (28.95, 36.70)	0.173
TBil (umol/L)	313.77 ± 129.44	378.95 ± 156.44	0.011
Cre (umol/L)	72.20 (61.00, 82.00)	76.00 (64.50, 86.50)	0.273
MELD	19.62 (17.77, 23.12)	22.59 (20.46, 25.27)	0.000
MELD-Na	17.22 (12.60, 20.53)	21.64 (17.56, 24.48)	0.001
iMELD	36.76 (33.25, 40.63)	41.66 (38.89, 45.66)	0.000

M: male; F: female; PLT: platelet; PT: prothrombin time; INR: international normalized ratio; AFP: alpha-fetoprotein; ALT: alanine aminotransferase; AST: aspartate aminotransferase; Alb: albumin; TBil: total bilirubin; Cre: creatine; MELD: model for end-stage liver disease; MELD-Na: MELD-sodium; iMELD: integrated MELD.

**Table 2:** Correlation of PLT with liver function parameters and MELD series scores.

Parameter	PLT	
	*r* Value	*p* Value
ALT	0.227	0.003
AST	0.084	0.278
TBil	0.015	0.847
AFP	0.063	0.413
Alb	0.219	0.004
PT	−0.073	0.347
INR	−0.057	0.461
MELD	−0.042	0.583
MELD-Na	−0.089	0.249
iMELD	−0.155	0.043

PLT: platelet; ALT: alanine aminotransferase; AST: aspartate aminotransferase; TBil: total bilirubin; AFP: alpha-fetoprotein; Alb: albumin; PT: prothrombin time; INR: international normalized ratio; MELD: model for end-stage liver disease; MELD-Na: MELD-sodium; iMELD: integrated MELD.

**Table 3:** Univariate and multivariate Cox regression analysis of 180-day mortality.

Parameter	β	Univariate HR (95% CI)	*p*	β	Multivariate HR (95% CI)	*p*
Age (years)	0.049	1.050 (1.020–1.081)	0.001	0.043	1.044 (1.012–1.077)	0.007
PLT (109/L)	−0.009	0.991 (0.984–0.997)	0.005	−0.009	0.990 (0.984–0.997)	0.021
PT (s)	0.004	1.004 (0.985–1.023)	0.672			
INR	0.463	1.589 (0.995–2.539)	0.053			
AFP (ng/mL)	−0.001	0.999 (0.997–1.000)	0.136			
ALT (IU/L)	0.000	1.000 (0.999–1.000)	0.148			
AST (IU/L)	0.000	1.000 (0.999–1.000)	0.416			
Alb (g/L)	−0.062	0.940 (0.875–1.011)	0.094			
Tbil (umol/L)	0.003	1.003 (1.001–1.005)	0.012	0.002	1.002 (1.000–1.004)	0.042
Cre (umol/L)	0.000	1.000 (0.986–1.021)	0.177			
MELD	0.029	1.029 (1.004–1.055)	0.021	0.006	1.006 (0.897–1.127)	0.310
MELD-Na	0.031	1.031 (1.010–1.053)	0.003	0.009	1.009 (0.928–1.097)	0.458
iMELD	0.034	1.035 (1.015–1.054)	0.000	0.142	1.037 (1.016–1.059)	0.032

PLT: platelet; PT: prothrombin time; INR: international normalized ratio; AFP: alpha-fetoprotein; ALT: alanine aminotransferase; AST: aspartate aminotransferase; Alb: albumin; TBil: total bilirubin; Cre: creatine; MELD: model for end-stage liver disease; MELD-Na: MELD-sodium; iMELD: integrated MELD.

**Table 4:** AUC and cut-off values of the prognostic variables.

ROC	AUC	95%CI	Sensitivity	Specificity	Youden Index	Cut Off Value	*p* Value
	Area						
PLT	0.643	0.539–0.747	61.00%	65.90%	0.269	99.50	0.006
MELD	0.695	0.609–0.782	82.90%	56.60%	0.395	20.20	0.001
MELD-Na	0.679	0.590–0.767	63.40%	73.60%	0.371	19.87	0.001
iMELD	0.745	0.666–0.823	75.60%	66.70%	0.423	39.30	0.001
PLT-iMELD	0.758	0.678–0.838	82.90%	62.00%	0.449	4.50	0.001

ROC: receiver operating characteristic curve; AUC: area under curve; CI: confidence interval; PLT: platelet; MELD: model for end-stage liver disease; MELD-Na: MELD-sodium; iMELD: integrated MELD; PLT-iMELD: platelet-integrated MELD.

## Data Availability

The data presented in this study are available on request from the corresponding author. The data are not publicly available due to patients are still being recruited and used in unconcluded funds.

## Abbreviations

PLT	Platelet
HBV-ACLF	Hepatitis B-related acute-on-chronic liver failure
PE	Plasma exchange
MELD	Model for end-stage liver disease score
ROC	Receiver operation characteristic
TBil	Total bilirubin
AUC	Area under the curve
ACLF	Acute-on-chronic liver failure
SMT	Standard medical treatment
ALSS	Artificial liver support system
LT	Liver transplantation
AFP	Alpha-fetoprotein
TPO	Thrombopoietin
PWR	PLT to WBC ratio
PT	Prothrombin time
INR	International normalized ratio
ALT	Alanine aminotransferase
AST	Aspartate aminotransferase
Alb	Albumin
Cre	Creatine
MELD	Model for end-stage liver disease
MELD-Na	MELD-sodium
iMELD	Integrated MELD
APASL	Asian Pacific Association for the Study of the Liver AARC APASL
CI	Confidence interval
5-HT	5-Hydroxytryptamine
